# HueFertility Ovulation: A Smartphone-Enabled Quantification System for Urinary Luteinizing Hormone in a Lateral Flow Assay

**DOI:** 10.3390/bios16100589

**Published:** 2026-10-20

**Authors:** Nidhi Menon, Erdi Kara, Prava Sharma, Adrian Crutchfield, Ayesha Azimuddin, Cianna Chung, Brianna Wronko-Stevens

**Affiliations:** HueDx, Inc., Philadelphia, PA 19104, USA; erdi.kara@huedx.com (E.K.); ayesha.azimuddin@huedx.com (A.A.); ciannachung@gmail.com (C.C.);

**Keywords:** luteinizing hormone, ovulation, hormone tracking, smartphone quantification, lateral flow, color correction, deep learning, equitable diagnostics

## Abstract

Luteinizing hormone (LH) is a critical biomarker for ovulation prediction and reproductive health monitoring, yet most commercially available at-home LH tests rely on qualitative or semi-quantitative visual interpretation, limiting their clinical utility. We present HueFertility Ovulation, a smartphone-enabled quantitative LH measurement system comprising a lateral flow immunoassay dipstick and a mobile application that integrates proprietary color correction algorithms with a deep learning regression model to deliver objective LH concentration estimates from strip images captured under variable real-world conditions. Color correction using the HueCard reference system substantially reduced inter-image color variability, decreasing mean ΔE00 from 11.19 to 4.02 across diverse lighting environments. When evaluated against known LH concentrations in a separately acquired independent dataset, the regression model achieved $R^{2}=0.92$ and a mean absolute percentage error (MAPE) of 17.4%. The experimental limits of blank, detection, and quantitation were 0.64, 3.18, and 10.00 mIU/mL, respectively. Multi-site precision testing demonstrated %CVs within 25% across three independent locations. At a per-test cost of $0.60 with no external hardware required, HueFertility offers a low-cost, accessible platform for quantitative LH monitoring, with utility in fertility planning, hormonal health tracking, and resource-limited settings. Ultimately, the study highlights the opportunity to extend the technology to other low-cost lateral flow tests and clinical chemistry applications.

## 1. Introduction

Luteinizing hormone (LH) is a glycoprotein hormone belonging to the gonadotropin family and plays a central role in the regulation of reproductive physiology in both males and females. It is secreted by the pituitary gland and plays an important role in controlling ovulation, menstrual cycles, and gonadal steroidogenesis in females [1]. In males, LH stimulates the Leydig cells of the testes to produce testosterone, which is essential for spermatogenesis and the development of secondary sexual characteristics [2].

Given its role in ovulation, LH has been widely adopted as a biomarker for fertility monitoring, particularly in women trying to conceive [3,4]. LH tests are low-cost, accessible, and have demonstrated utility in increasing pregnancy rates by guiding the timing of intercourse. However, the vast majority of commercially available LH tests are qualitative or semi-quantitative in nature, relying on visual interpretation of color intensity or subjective comparison of test and control lines. As a result, these assays provide limited insight into absolute LH concentrations or dynamic changes in hormone levels across the menstrual cycle.

Beyond its established role in ovulation, dysregulation of LH secretion and signaling is implicated in a range of reproductive and systemic pathologies that are often under-recognized in routine clinical and consumer testing. In females, anomalies in LH production can lead to disorders such as polyendocrine metabolic ovarian syndrome (PMOS), luteal phase defects, and infertility [5]. In males, disruption of normal LH production due to pituitary dysfunction, genetic or other factors can lead to infertility, hypogonadism, testicular atrophy, and other physiological dysfunctions [6].

Despite the growing recognition of LH’s broader clinical relevance, current at-home LH testing platforms remain limited by subjective interpretation and lack of quantitative resolution. Visual assessment of lateral flow test strips is inherently prone to user variability, lighting conditions, and differences in baseline LH levels between individuals. These factors can contribute to an incorrect interpretation of the results, particularly in populations with atypical hormonal profiles, such as women with PMOS or individuals near perimenopause and menopause [7,8]. Previous studies have demonstrated that quantitative measurement of urinary LH improves the accuracy of ovulation prediction and the identification of the fertile window, supporting the need for more objective and sensitive testing methodologies [9].

To address these limitations, we developed a urinary LH dipstick lateral flow assay coupled with a smartphone-enabled quantitative analysis platform, comprising HueFertility, an application for imaging and quantifying strips. By integrating HueDx’s proprietary color correction algorithms, as well as the LH calibration model on the back-end, HueFertility enables robust quantification of LH concentrations from colorimetric test strip images captured under variable real-world conditions [10]. This approach aims to improve analytical sensitivity, reduce user interpretation bias, and expand inclusivity and clinical relevance of at-home LH testing, providing a foundation for reproductive and hormonal health monitoring. To determine the performance characteristics of the system, we evaluated the precision, detection limits, interference, native sample validation, and exploratory comparison of quantitative competitor strips available on the market against the gold-standard ELISA.

## 2. Materials and Methods

### 2.1. Luteinizing Hormone Test Strip Development

Luteinizing hormone test strips (sandwich immunoassay) were developed using mouse anti-LH matched-pair monoclonal antibodies for capture and detection, and goat anti-mouse IgG for control line striping. The detection antibody was conjugated with colloidal gold (40 nm) and coated on the conjugate pads. The strips were cut to 3 mm width, individually packaged with silica gel desiccants and heat-sealed. The strips were manufactured by Levram LifeSciences (Silvassa, India) according to specifications provided by HueDx, Inc. (Philadelphia, PA, USA).

### 2.2. Lateral Flow Test HueCard and Sticker Manufacturing

The HueCard is a customizable platform consisting of a color sticker based on the original color-rendition chart, with some modifications to extend the range of brightness [10,11]. A lateral flow HueCard was designed using SolidWorks, and a mold was developed with the manufacturer (Levram LifeSciences, Silvassa, India) for scaling production. The color sticker was designed by HueDx, Inc. and manufactured to specifications by StickyLife (Jacksonville, FL, USA). In prior work, this approach was shown to substantially reduce inter-image color variability and improve downstream concentration prediction accuracy and reproducibility [10]. To evaluate manufacturing reproducibility, 10 distinct HueCards were imaged under identical lighting and imaging conditions using an iPhone 13 (Apple Inc., Cupertino, CA, USA). For each color patch, pairwise color differences were computed across all card pairs (45 comparisons per patch) using the CIE ΔE00 metric. To evaluate robustness to illumination, a single HueCard was imaged under multiple lighting conditions. For each color patch, ΔE00 values were computed both within each lighting condition (across replicate images) and across different lighting conditions. In addition, ΔE00 values relative to the ground-truth reference colors were computed for each patch before and after color correction.

### 2.3. System Calibration

In order to quantify LH concentrations, we calibrated the test strips on the lateral flow HueCard. Luteinizing hormone (WHO Standard 81/535, NIBSC, Potters Bar, UK) was used to spike normal pooled male urine (Golden West, OH2000, Temecula, CA, USA) to create the calibration solutions. Male urine was chosen because it was closest to the intended female urine biological matrix, with negligible levels of interfering hormones that may otherwise be present in female urine. An FDA registered In Vitro Diagnostic LH Urine ELISA kit (Alpco, 20-LHUHU-E01, Salem, NH, USA) was used to determine the baseline LH concentrations in the male control urine sample and male urine samples spiked with LH. Optical density (OD) for ELISA was read at 450 nm and background was subtracted at 620 nm, using a BioTek Synergy Neo 2 (Winooski, VT, USA) plate reader. Strips were dipped in urine up to the max line for 20 s, after which they were laid on the HueCard to develop. After 8 min, the strips were ready to be imaged. Images of unique strips were captured from 8 min to 10 min using HueTools [12].

### 2.4. Image Processing and Predictive Model Development

#### 2.4.1. Image Acquisition

Following assay calibration, we collected a structured image dataset to capture variability arising from manufacturing, image acquisition, and capture timing. Lateral flow strips corresponding to known LH concentrations were imaged across multiple locations and smartphone devices under controlled acquisition conditions. This dataset serves as the input to the downstream image processing and modeling stages. All images used for model training and evaluation in this study were processed using the HueDx image registration and color normalization pipeline prior to region-of-interest extraction and regression modeling. Color normalization was performed using the HueCard reference system, a proprietary printed color reference developed and validated prior to use. Table 1 and Table 2 summarize the training, model-selection validation, and independent evaluation sets and their distributions across concentration levels, phones, and acquisition locations.

#### 2.4.2. ROI Extraction

Following image registration and color normalization, a region of interest (ROI) encompassing the full vertical assay area was automatically extracted. This step was formulated as an object detection problem, in which the assay region was localized based on learned spatial and visual features of the strip in the well. The extracted ROI includes the test line, control line, and surrounding background, providing a consistent spatial context for downstream analysis. In parallel, additional detection models were used for automated quality control, including identification of blank assays and verification of control line presence. These tasks were also framed as object detection problems, targeting visually well-defined patterns that distinguish valid from invalid assay images. Images identified as blank or lacking a detectable control line were excluded from further analysis, and no concentration prediction was returned for these cases. Because these conditions exhibit consistent and well-defined visual signatures, they were detected with near-perfect accuracy in our internal validation as well as production use.

#### 2.4.3. Predictive Model Development

We framed concentration estimation as a supervised image regression problem: the input is the extracted ROI image described above, and the output is a scalar LH concentration value. Model training therefore seeks to learn a direct mapping from assay appearance to concentration. The modeling dataset was partitioned separately at each concentration into training and validation sets at the physical-strip level, ensuring that all images of a strip remained in one partition. The validation set was used for learning-rate scheduling, checkpoint selection, and early stopping. After model development, performance was assessed on a separately acquired independent evaluation set that was not used for model fitting, hyperparameter selection, early stopping, or checkpoint selection. The numbers of physical strips and images are reported in Table 1; Table 2 gives the corresponding image distributions by phone and acquisition location. All images in the training, validation, and independent evaluation datasets were obtained using strips from a single reagent lot.

The regression model was implemented and trained in PyTorch 2.10 [13] using a transfer learning architecture based on MobileNetV3-Small [14], initialized with ImageNet weights, followed by a lightweight regression head. Input ROI images were resized to 224 × 224 pixels and normalized using ImageNet channel statistics prior to model input. Backbone features were globally pooled and passed through a fully connected regression head consisting of a 512-unit hidden layer with ReLU activation followed by a single scalar output. Concentration labels were transformed as log(1+c) during training to reduce the effect of dynamic-range imbalance; zero-concentration labels therefore remained zero. Predictions were back-transformed to the original mIU/mL scale for reporting. The model was trained end-to-end using Adam with Smooth L1 loss, an initial learning rate of $5\times 10^{-4}$, a batch size of 32, and a maximum of 300 epochs.

Random top-to-bottom flipping was applied only during training to improve robustness to the plausible user error of placing a strip upside down despite the provided placement instructions. Mild blur and brightness/contrast augmentation was also confined to training images. Validation images were evaluated without augmentation, and validation mean absolute error controlled learning-rate scheduling, checkpoint selection, and early stopping.

Model performance was reported on the independent evaluation set using standard regression metrics (R^2^, MAE, RMSE, and MAPE; with MAPE computed on non-zero targets), along with concentration-level summaries of mean prediction, standard deviation, precision, and total error. Precision was reported as %CV, computed from the standard deviation of replicate predictions relative to their mean prediction, and %TE was computed using an RMS formulation combining bias and imprecision. Model-derived analytical sensitivity metrics were also calculated from the independent evaluation predictions: LoB was estimated from the distribution of predictions at zero concentration, LoD from LoB and the imprecision at the lowest non-zero concentration, and LoQ as the lowest concentration meeting a total error threshold of 20% [15].

We should note that preliminary experiments (not presented here) with regression models based on hand-crafted color features were also conducted; however, these approaches yielded lower predictive performance compared to the deep learning model, motivating the use of a deep learning approach.

### 2.5. HueFertility Smartphone Application

HueFertility leverages Apple iPhone software/hardware technologies to capture, process, and analyze images using computer vision (CV), machine learning (ML), and artificial intelligence (AI) to deliver qualitative and quantitative results in real time. Using the Swift programming language (Apple’s native language), we make use of Core Frameworks API to access and optimize iPhone hardware for raw sensor imaging under variable lighting conditions. The system employs advanced computational photography techniques to enhance image fidelity, ensuring accurate colorimetric analysis. After image capture, the data is securely transmitted to HueFertility backend cloud services to process the image through the aforementioned pre-processing pipelines.

### 2.6. Measurement of Performance Characteristics

Luteinizing hormone testing is exempt from regulatory approval. However, further experimental performance characteristics were measured using modified clinical laboratory standards guidelines to determine precision across the range, detection limits, interference, validation with female urine matrix and comparison to competitors [15,16,17]. All studies were carried out with contrived urine samples spiked with luteinizing hormone (WHO Standard 81/535, NIBSC), except the validation with native and spiked female urine. All studies were run with the HueFertility Ovulation kit (HueDx, Inc., Philadelphia, PA, USA).

#### 2.6.1. Precision

Five samples were tested in three unique locations, with unique iPhones (iPhone 11, iPhone 16 Pro, and iPhone 17) in each location. Each sample was tested five times in each location, and the strips were quantified using the HueFertility application. Concentrations were noted and the mean and percentage coefficients of variation were calculated. An average CV threshold of 25% was chosen based on the clinical relevance of deviations and the variability observed in manufacturing lateral flow tests at scale.

#### 2.6.2. Detection Limits

Four blank samples (no LH present) and four known low concentrations of LH (1.25, 2.5, 5, and 10 mIU/mL) were used to determine limit of blank (LoB) and limit of detection (LoD), respectively. Two readers were used for both studies, and each blank/low sample was run thrice for each reader. A non-parametric method was used to determine the limit of blank (LoB). The LoB of the measurement procedure was the maximal value of the LoBs obtained for each reader. Parametric analysis was used to determine limit of detection (LoD). Similar to the LoB, the LoD of the measurement procedure was the maximal value of the LoDs obtained for each reader. The %TE criterion was set at 30% for the experimental limit of quantitation (LoQ).

#### 2.6.3. Interference

Follicle-stimulating hormone (FSH), thyroid-stimulating hormone (TSH), and beta-human chorionic gonadotropin (β-hCG) were tested to determine interference levels. Charcoal-stripped female urine (BioIVT, Westbury, NY, USA) samples were spiked to produce six total samples each for FSH, TSH and β-hCG studies. Charcoal-stripped specimens are free from endogenous hormones, steroids, and metabolites that may cause unknown interferences. Three test samples of each hormone were prepared with spiked urine (FSH (50 mIU/mL), TSH (0.004 mIU/mL), β-hCG (25 mIU/mL) with three LH levels (blank, low, high). Control samples for each study were produced with three LH levels (blank (0 mIU/mL), low (10 mIU/mL), high (50 mIU/mL)), replacing the interferent with distilled water. Ten replicates were run for each sample of the interferent.

#### 2.6.4. Validation with Native Female Urine Samples

Further validation with the intended matrix was carried out with 10 unique female urine samples (BioIVT). In addition, all samples were spiked with LH to introduce 10 additional samples to cover the quantitative range of the product. While the use of spiked samples is generally not recommended due to the potential for inaccurate results, it is an acceptable practice when samples cannot be procured for the full quantitative range. All 20 samples used in the study were tested using a validated ELISA (Alpco, 20-LHUHU-E01) and with the HueFertility Ovulation kit (HueDx, Inc., Philadelphia, PA, USA). Each sample was run in triplicate across both methods. A Deming regression analysis was carried out across the native and spiked samples.

#### 2.6.5. Competitor Comparison Study

The analytical performance of HueFertility was compared with three quantitative urinary LH consumer tests using a validated ELISA kit (Alpco, 20-LHUHU-E01) as the reference method across a range of urinary LH concentrations. The devices represented distinct measurement methods: Competitor 1 is a smartphone-enabled lateral flow reader requiring no external hardware, representing the most analogous technology to HueFertility; Competitor 2 employs a fluorescence-based external hardware reader; and Competitor 3 is a smartphone-enabled reader incorporating a device-specific optical attachment to control for ambient lighting conditions. A total of four samples were prepared for this study. Competitor 1 (n = 2) and HueFertility (n = 3) were run using different phones in different locations. Competitor 2 (n = 3) did not require a phone and was run with a stand-alone reader and the values were recorded. Competitor 3 (n = 3) was run using one phone with the attachment in a single location since ambient lighting did not influence readout. A contributing factor to the limited number of replicates for competitor devices was the per-user test restrictions enforced by competitor applications: these platforms impose limits on the number of tests a single user account can run consecutively, which inhibited repeated testing without registering additional user accounts. This practical constraint is acknowledged as a limitation of the design of the comparison study. Deming regression analysis was applied to each method against ELISA.

## 3. Results

### 3.1. HueCard Color Correction Improves ΔE00 Scores for Reliable Quantification of Lateral Flow Assays

To reduce variability in smartphone-based image measurements, we applied a color correction step that standardizes each image using the known reference colors on the HueCard. This process minimizes differences arising from ambient lighting, camera characteristics, and capture conditions, ensuring that the observed color intensities of the lateral flow assay are comparable across images. Because quantitative analysis relies on accurate measurement of test and control line intensities, uncorrected variation in color rendering can introduce significant error. By normalizing each image to a common visual standard prior to analysis, color correction improves the reliability and consistency of downstream quantification. We quantify this improvement using the ΔE00 color difference metric, a widely used measure of perceptual color error, where lower values indicate closer agreement between measured and reference colors; values below approximately 5 are generally considered to be near or below the threshold of perceptible color difference. A direct comparison of model performance using images before and after color correction was reported in our prior work [10]; further methodological details are provided in [10,12].

The color sticker is applied to the plastic mold to produce the physical HueCard. The HueCard color sticker consists of twenty-six reference colors, as shown in Figure 1A. Figure 1B shows a representative HueCard with an LH lateral flow strip (left) and its color- corrected counterpart (right), normalized to a standard illuminant condition. To assess manufacturing reproducibility, we analyzed 10 distinct HueCards imaged under identical acquisition conditions. For each color patch, we computed pairwise ΔE00 values across all card pairs (45 comparisons per patch), and summarized these values by their mean and maximum. The metrics reported represent the average of per-patch means and the overall maximum across all patches. As shown in Figure 1C, the mean and maximum ΔE00 values before correction were 1.37 and 4.80, respectively, indicating low variability consistent with expected printing tolerances. After color correction, the mean ΔE00 decreased to 0.68 and the maximum to 3.75, demonstrating that the correction pipeline further reduces residual card-to-card color variation.

To evaluate the impact of illumination on measurement reliability, we analyzed a single HueCard imaged under four distinct lighting conditions (approximately 2700 K, 3000 K, 5500 K, and 6500 K), with multiple replicate captures per condition. We first assessed intra-site consistency, defined as the repeatability of measurements under fixed illumination. For each lighting condition and color patch, pairwise ΔE00 values were computed across replicate images and aggregated per patch. As shown in Figure 2A,B, intra-site variability was low across all conditions and further reduced after color correction, indicating improved repeatability of color measurements. We next evaluated inter-site consistency, which captures variability introduced by changes in illumination. For each color patch, mean patch colors from all images across the four lighting conditions were compared using pairwise ΔE00 values and aggregated across patches. As shown in Figure 3A,B, substantial variability was observed prior to correction, reflecting the strong influence of lighting differences on measured color. After color correction, this variability was markedly reduced, demonstrating improved robustness of the system to changes in illumination. Finally, we assessed absolute color accuracy by comparing measured patch colors to their known reference values on the HueCard. For each image and color patch, ΔE00 values were computed relative to the ground-truth reference color and summarized across lighting conditions. As shown in Figure 4A,B, the mean ΔE00 decreased from 11.19 before correction to 4.02 after correction, with corresponding reductions in maximum error (17.05 to 7.29) and variability (standard deviation 3.95 to 1.91). These results indicate that color correction substantially improves both the accuracy and consistency of color measurements across diverse illumination environments.

### 3.2. Calibration of Luteinizing Hormone Strips

Raw smartphone images of the assay are first registered to a common reference frame using the HueCard for consistent spatial alignment across samples. Registered images are then color-normalized to reduce variability. Finally, a vertical region of interest (ROI) encompassing the test line, control line, and surrounding background is extracted and used as input for downstream regression modeling. On the 99 images in the separately acquired independent evaluation set, individual model predictions yielded $R^{2}=0.92$, a mean absolute error of 6.39 mIU/mL, and an RMSE of 9.37 mIU/mL against known LH concentrations (Figure 5). Averaged across the non-zero concentration levels, the percentage coefficient of variation (%CV) was 18.4%, percentage total error (%TE) was 21.7%, and mean absolute percentage error (MAPE) was 17.4%. Model-derived estimates calculated from these independent evaluation predictions were 1.1 mIU/mL for the limit of blank (LoB), 2.3 mIU/mL for the limit of detection (LoD), and 5.0 mIU/mL for the limit of quantitation (LoQ), using the specified 20% total-error criterion. These model-derived estimates are distinct from the experimental measurement-procedure limits reported in Section 3.4.

### 3.3. HueFertility Workflow

The application was developed to be HIPAA compliant, ensuring identifiable data was not sent to the cloud and test images could not be traced back to a user. The workflow involves signing in and first scanning the QR code on the back of the HueCard for assay traceability. The application then guides the user through the process of dipping the test strips, including a timer to ensure consistency across user testing. Once the test is complete, the application guides image capture using a traceable HueCard guide to align with the HueCard. An image is captured automatically when the outline on the camera perfectly aligns with the HueCard to be imaged. Following image capture, the application refreshes to the Result page, which also includes a chart to track hormone values over a period of time. Supplementary Video S1 shows the HueFertility workflow simultaneously with the testing process.

### 3.4. Performance Characteristics

Precision testing was carried out across three locations, with unique iPhone readers at each location. Real-world testing showed %CVs under 25% for all concentrations. Figure 6A shows the distribution of the HueFertility quantitative readouts across the three locations. Limit of blank (LoB) was determined using the non-parametric method for ranking quantitative readouts independently across the two phone readers. The rank position was calculated based on CLSI guidelines in EP17-A2 [15], and LoB was calculated to be 0.64 mIU/mL. Limit of detection (LoD) followed the parametric methods outlined in the CLSI guidelines in EP17-A2. The LoD of the measurement procedure is the higher value of the LoDs obtained for each phone reader tested. This was calculated to be 3.18 mIU/mL. Figure 6B shows the distribution of results for the blank and low-level samples used in LoB and LoD calculations. LoQ was determined using %TE criterion under 30%, which was first acceptable at 10 mIU/mL. Interference testing had mean differences between control and spiked samples under 2 mIU/mL for the blank, 8 mIU/mL for the low, and 10 mIU/mL for the high luteinizing hormone urine samples. Table 3 shows the mean difference between the control samples and the spiked interference samples.

Validation studies with the intended female urine matrix using ELISA and HueFertility Ovulation were carried out. Deming linear regression analysis, shown in Figure 7, was carried out, and a slope of 1.36 (95% CI 1.12 to 1.59) and intercept of 0.45 (95% CI −1.80 to 2.70) were calculated. Individual slopes were similar for native (1.25) and spiked (1.34) samples.

Exploratory measurement comparisons were made using ELISA and three competitor devices claiming quantitative readouts. Deming regression analysis (Figure 8) shows the readings across the devices. All devices showed a linear relationship with ELISA. HueFertility and Competitor 2 showed proportional positive bias: HueFertility had a slope of 1.49 (95% CI 1.04–1.94) and Competitor 2 a slope of 1.48 (95% CI 1.37–1.60). Competitor 2 also showed a significant negative constant bias (intercept −4.36, 95% CI −7.80 to −0.93), whereas HueFertility did not (intercept 3.00, 95% CI −6.65 to 12.64). Competitor 1 showed no statistically significant proportional or constant bias relative to ELISA (slope 1.06, 95% CI 0.41–1.71; intercept 5.08, 95% CI −11.55 to 21.70); however, the wide confidence intervals reflect the limited sample size and indicate that clinically relevant bias cannot be excluded. Competitor 3 showed significant proportional negative bias relative to ELISA (slope 0.55, 95% CI 0.43–0.66), with no significant constant bias (intercept −0.19, 95% CI −2.93 to 2.55).

## 4. Discussion

Smartphone-enabled platforms can facilitate longitudinal self-tracking, enable earlier detection of abnormal hormonal trends, and support more informed clinical discussions, particularly in settings where access to laboratory testing is limited. This may also reduce disparities in reproductive healthcare by providing objective, low-cost, and user-friendly diagnostics that are adaptable to diverse populations. This study reports the analytical characterization of HueFertility, a smartphone-enabled consumer lateral flow reader for the quantitative measurement of urinary luteinizing hormone (LH), benchmarked against a validated ELISA reference method and three commercially available quantitative LH monitoring devices. Taken together, the data demonstrate that HueFertility provides LH quantification across a clinically relevant concentration range, with acceptable precision, well-defined analytical limits, and minimal susceptibility to hormonal interferents at clinically relevant surge thresholds, at a substantially lower cost of use than competitor devices.

The color correction methods used with the optimized color sticker for this assay demonstrated a significant improvement in ΔE00 values for both intra-site and across various ambient lighting conditions, as shown in Figure 1, Figure 2, Figure 3 and Figure 4. On the independent evaluation set spanning 0–100 mIU/mL, HueFertility predictions showed a coefficient of determination of $R^{2}=0.92$ and a MAPE of 17.4% relative to the known LH concentrations. Mean predictions tracked the line of identity across the evaluated range, with greater dispersion at some concentration levels consistent with the expected behavior of lateral flow immunoassay platforms. Multi-site precision was assessed across three independent testing locations using five samples across the clinical LH range. The coefficient of variation (%CV) ranged from 1.79% at the highest concentration (mean 65.51 mIU/mL) to 21.30% at a mid-range concentration (mean 42.27 mIU/mL), with all samples remaining within 25% CV. The observed inter-site variability is important in the context of a smartphone-based reader, where ambient lighting conditions, device camera characteristics, and user technique may differ between testing environments. The %CV within acceptable limits across all three locations suggests that the HueFertility optical system is sufficiently robust to accommodate real-world variability in testing conditions, a finding with direct relevance to the end-user’s test setting.

The experimental analytical limits of blank, detection, and quantitation were determined as 0.64, 3.18, and 10.00 mIU/mL, respectively. The LoQ of 10.00 mIU/mL is particularly relevant in the context of the intended clinical application. The established LoQ falls below the normal surge threshold, indicating that HueFertility is capable of quantifying LH at concentrations relevant to both baseline monitoring and surge detection, a prerequisite for meaningful cycle tracking [18]. The LoD of 3.18 mIU/mL provides a further margin of sensitivity below the LoQ, ensuring that very low LH values can be distinguished from background noise. The LoB of 0.64 mIU/mL confirms that blank matrix does not generate signal at a level that would confound interpretation of low-concentration samples.

The susceptibility of the strips to hormonal interferents was evaluated by assessing the mean difference in LH readings between control samples and samples spiked with follicle-stimulating hormone (FSH, 50 mIU/mL), thyroid-stimulating hormone (TSH, 0.004 mIU/mL), and human chorionic gonadotropin (hCG, 25 mIU/mL) at blank, low (10 mIU/mL), and high (50 mIU/mL) LH concentrations (Table 3). TSH at 0.004 mIU/mL produced minimal mean differences across all three LH concentration levels (blank: −0.014; low: 5.40; high: −0.044 mIU/mL), consistent with the structural dissimilarity between TSH and LH and the expected specificity of the anti-LH antibody system. FSH at 50 mIU/mL, a concentration substantially exceeding typical urinary FSH levels in the follicular phase [19], produced mean differences of 0.01, 8.07, and 4.66 mIU/mL at blank, low, and high LH concentrations respectively. β-hCG, at 25 mIU/mL, produced mean differences of 1.15, −7.33, and 0.50 mIU/mL at blank, low, and high concentrations of LH, respectively. The difference at low LH concentrations is noteworthy given the structural homology between hCG and LH, and may reflect partial cross-reactivity at concentrations where the LH signal itself is near the LoQ [20]. Clinically, urinary hCG at 25 mIU/mL would correspond to early pregnancy, a context in which LH surge monitoring is typically not the primary objective, and this limitation is therefore unlikely to affect the majority of intended use cases [20]. At 50 mIU/mL, near the clinical decision point, all mean differences were within ±20%, indicating no clinically significant interference [21]. Additionally, further testing using 10 native urine samples (in the 1–12 mIU/mL range) supported validation of native samples at low LH concentrations with unknown interferents.

At a per-test cost of 60 cents, and no requirement for expensive smartphone attachments or external hardware readers, HueFertility offers a low-cost, reliable method of quantification based on performance. Table 4 summarizes the performance characteristics of the various strips available in the market. HueFertility has the fastest testing time (8 min), zero hardware cost, and the lowest per-test cost. However, the slope of Competitor 3 relative to ELISA was surprisingly low, given that a prior study reported its calibration against ELISA [22]. That study calibrated using six known concentration samples, but did not provide the LH reference standard used for calibration or the ELISA standards used in their comparison study. Overall, these findings have practical implications for accessibility. The financial barrier associated with dedicated reader hardware and high per-test consumable costs may limit sustained use of Competitors 2 and 3 in price-sensitive populations. Competitor 1 warrants additional specific comment among the devices since it is the only other comparator test offering smartphone-enabled quantitative readout without external hardware. Its values appeared to plateau at 45 mIU/mL, with readings no longer increasing despite continued elevation of ELISA reference concentrations above this threshold. This would predictably compress the regression slope, independent of any inherent imprecision in the platform at lower concentrations. Therefore, higher concentration levels beyond this limit were not used in the study. Whether this plateau reflects a hard upper limit of quantification built into their algorithm, saturation of the assay’s dynamic range, or a deliberate calibration design choice is not possible to determine from our data.

From these preliminary performance data, HueFertility’s smartphone-based architecture eliminates hardware cost while maintaining quantitative performance within acceptable limits, representing a meaningful advance in the accessibility of quantitative lateral flow monitoring technology.

A few limitations of the present study should be acknowledged. The comparison study was conducted with a small sample set, which constrains the statistical power of the regression analysis. This renders the regression estimates less stable and more sensitive to individual data points, relative to the larger, more densely sampled calibration dataset. This might explain the deviation from unity observed for HueFertility in the comparison studies. As a result, the data presented should be treated as preliminary indicators of performance and not as definitive. Additionally, the interference study did not apply any thresholds due to a lack of established regulatory criteria. The descriptive nature of the interference analysis precludes conclusions about statistical significance. Presently, the application is only supported on iPhone 11 and later models. Current efforts focus on Android expansion to improve accessibility. Notwithstanding these limitations, the data presented here provide an initial characterization of the analytical performance of HueFertility Ovulation tests and establish a basis for comparative evaluation against existing consumer LH monitoring technologies. The combination of proportional accuracy, multi-site precision within acceptable limits, a clinically relevant LoQ, and a low cost of use positions HueFertility as a viable quantitative LH monitoring tool for population-level as well as resource-limited applications where existing higher-precision devices remain inaccessible.

## Figures and Tables

**Figure 1:**
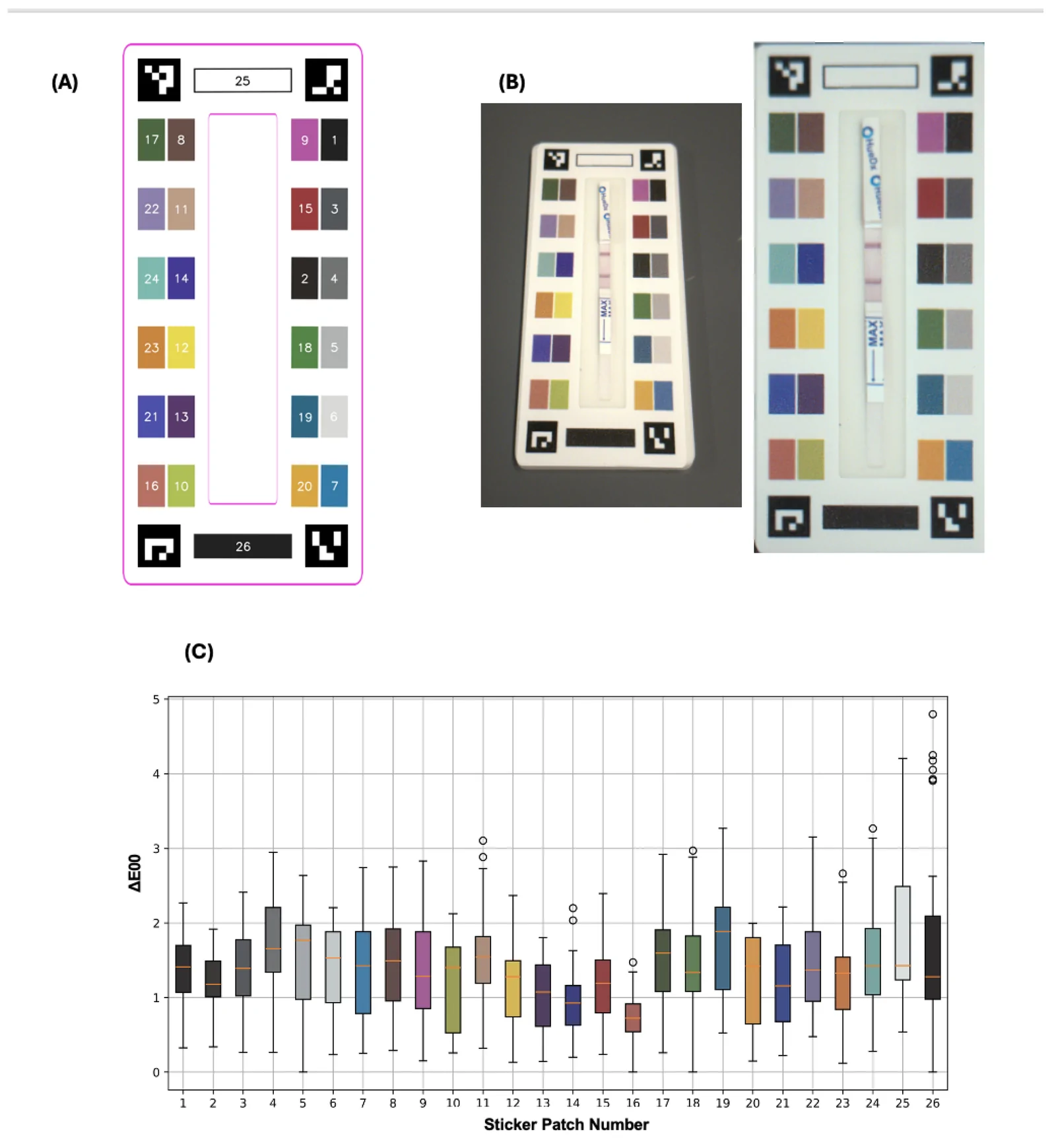
(**A**) HueCard template with indexed color chips. (**B**) HueCard raw assay image (**left**) and its color-corrected image under standard illuminant condition (**right**). (**C**) Mean ΔE00 scores of stickers received from the manufacturer.

**Figure 2:**
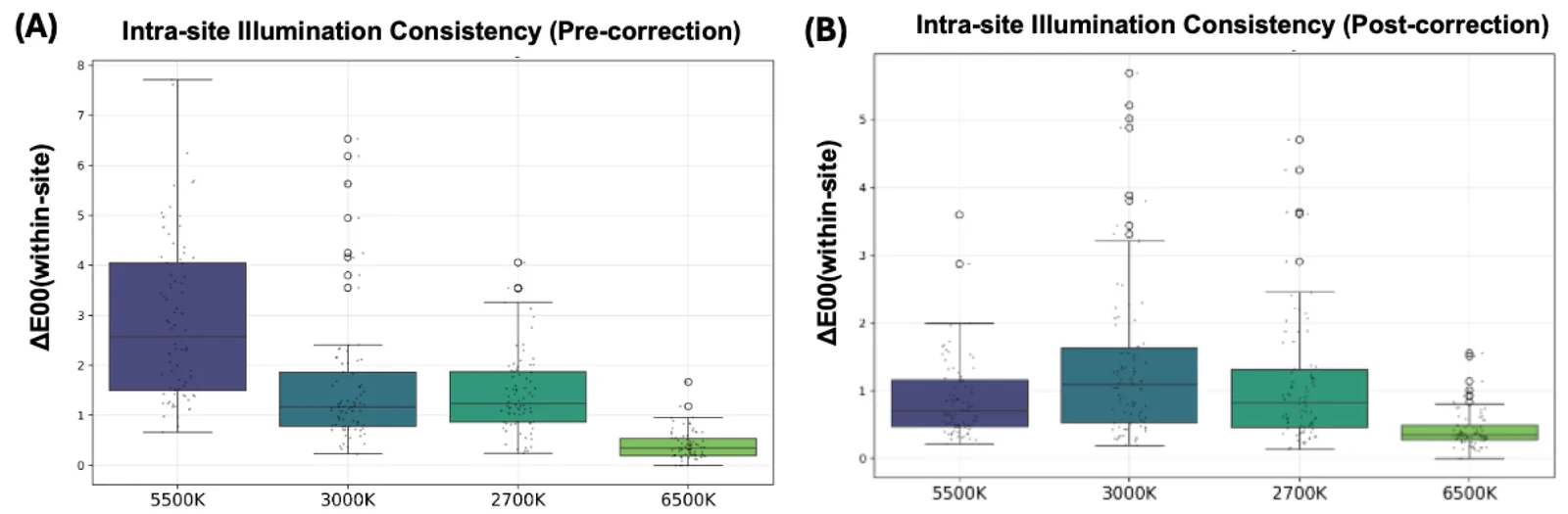
Intra-site consistency, showing pairwise ΔE00 distributions for replicate images acquired under identical illumination conditions before (**A**) and after (**B**) color correction.

**Figure 3:**
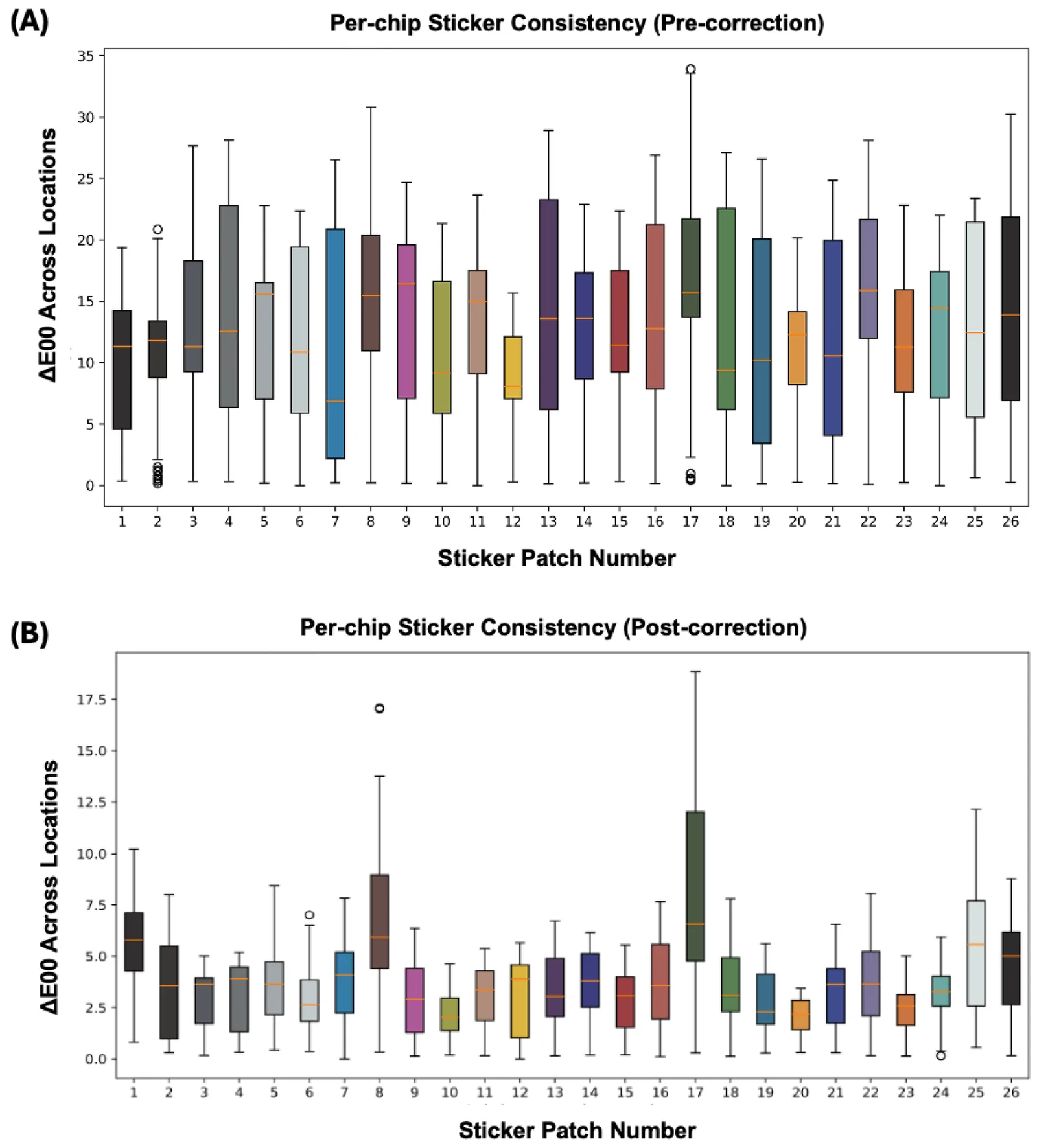
Inter-site consistency, showing variability of color patches across different illumination conditions before (**A**) and after (**B**) correction.

**Figure 4:**
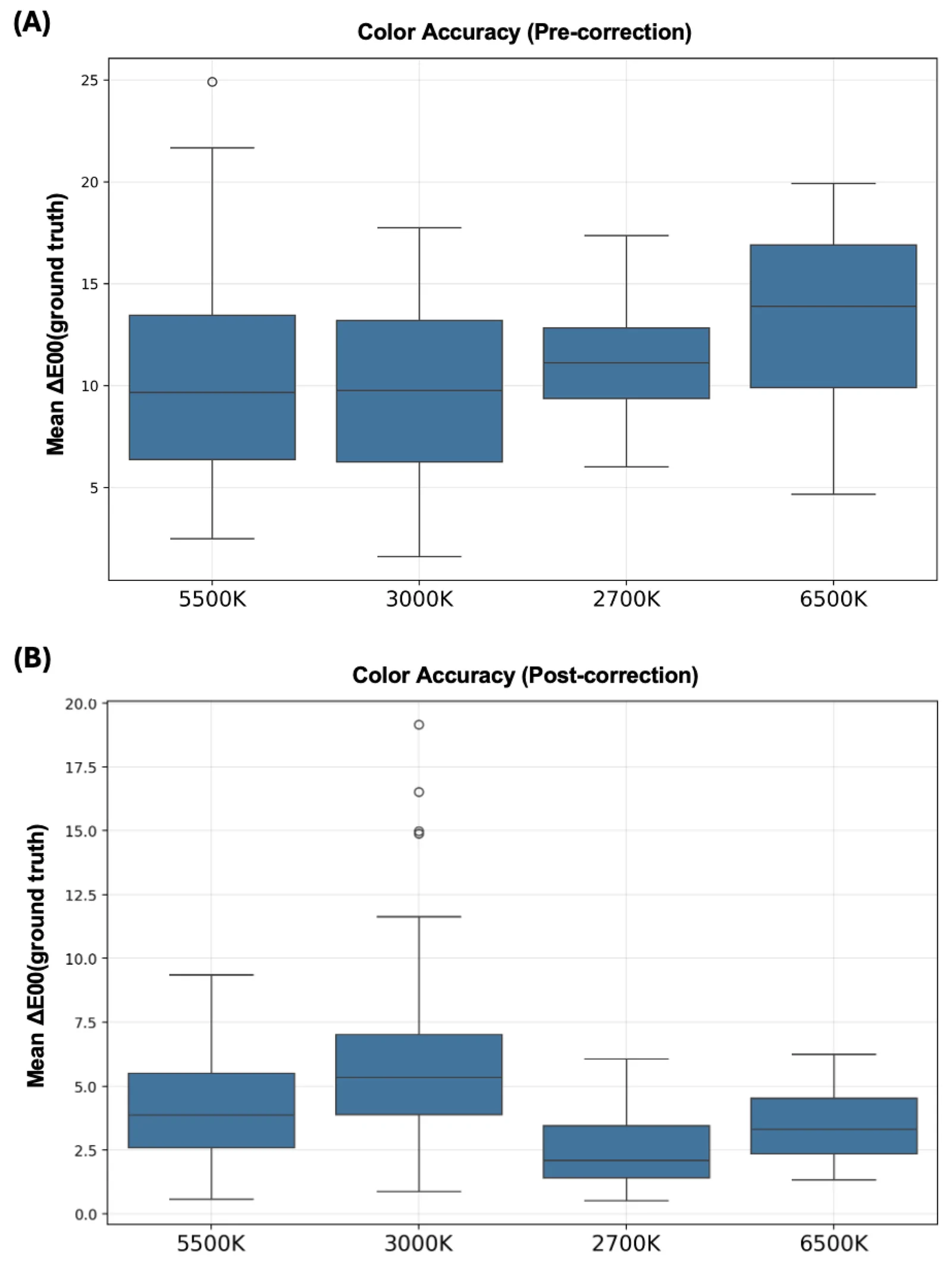
Color accuracy relative to ground-truth reference colors across lighting conditions (**A**) before and (**B**) after correction.

**Figure 5:**
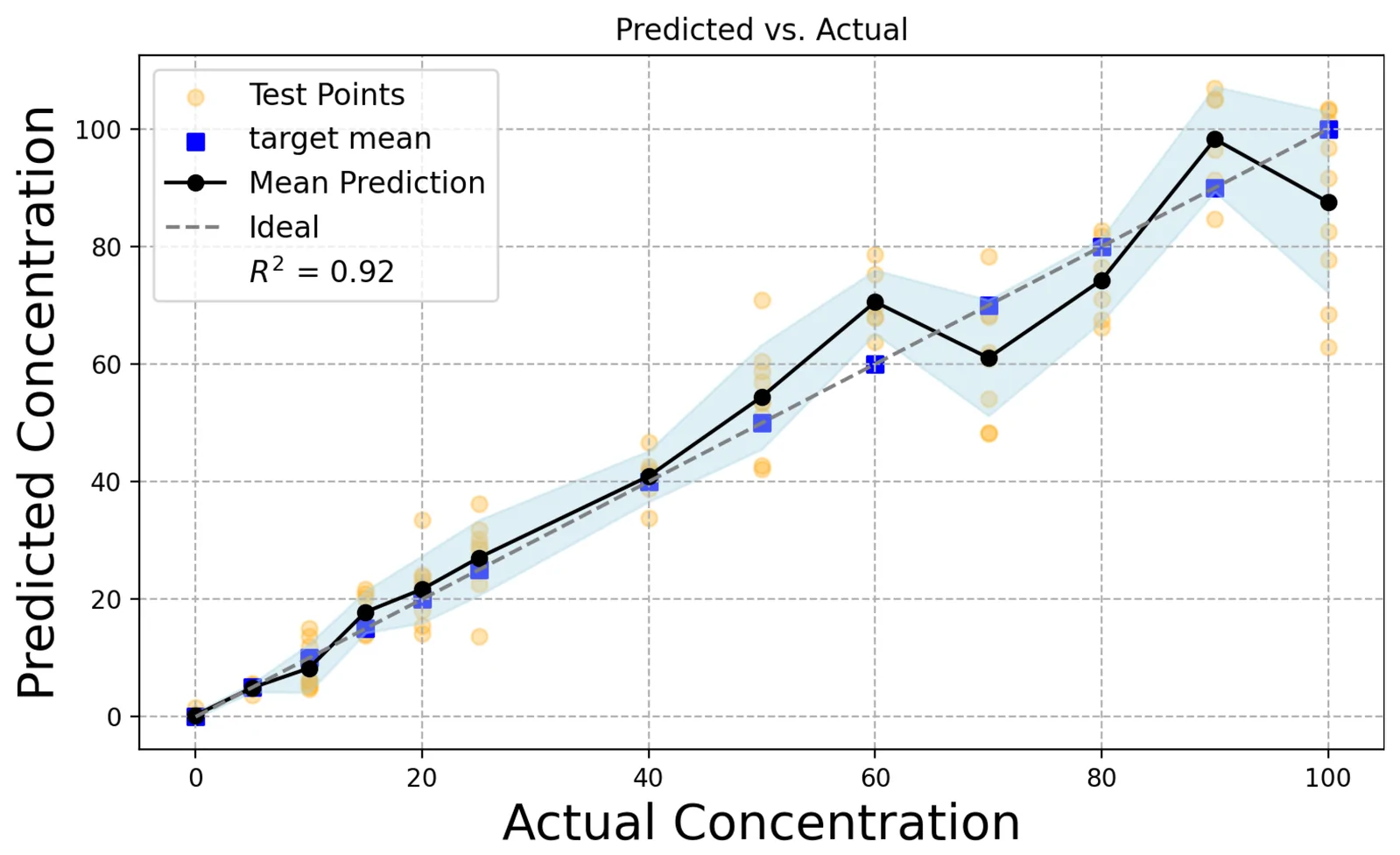
Scatter plot of model-predicted concentrations versus ground-truth values across the independent evaluation samples. Individual measurements are shown as points, with mean predictions at each concentration level overlaid. The dashed line represents ideal agreement (y=x). The model demonstrated strong correlation with ground truth ($R^{2}=0.92$) across the evaluated range.

**Figure 6:**
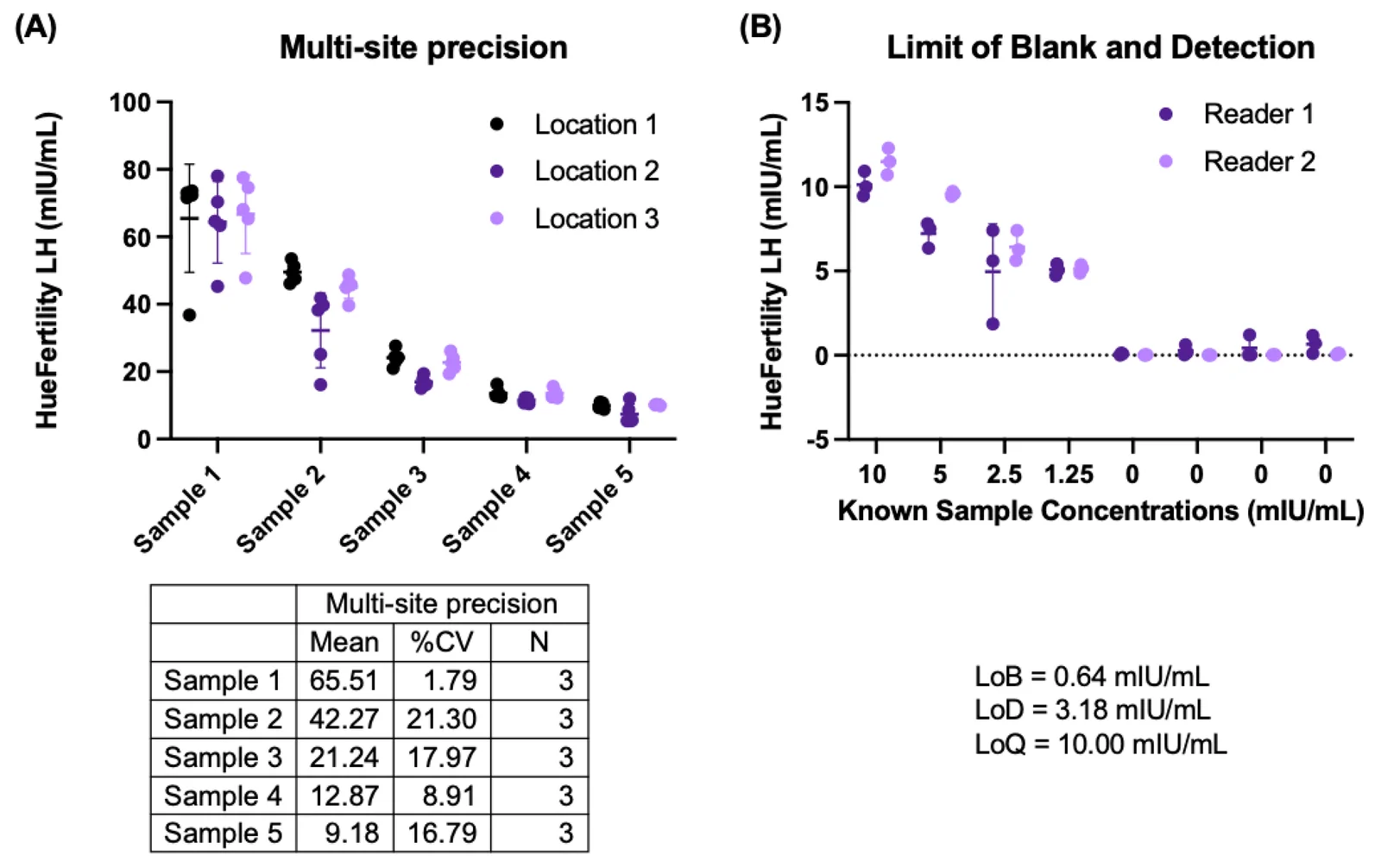
Precision and analytical limits. (**A**) Individual values plotted for each sample tested across three different locations. Bars display the mean ± SD. (**B**) Individual values plotted for each sample tested across two readers for each known blank and low-level sample. Bars display the mean ± SD.

**Figure 7:**
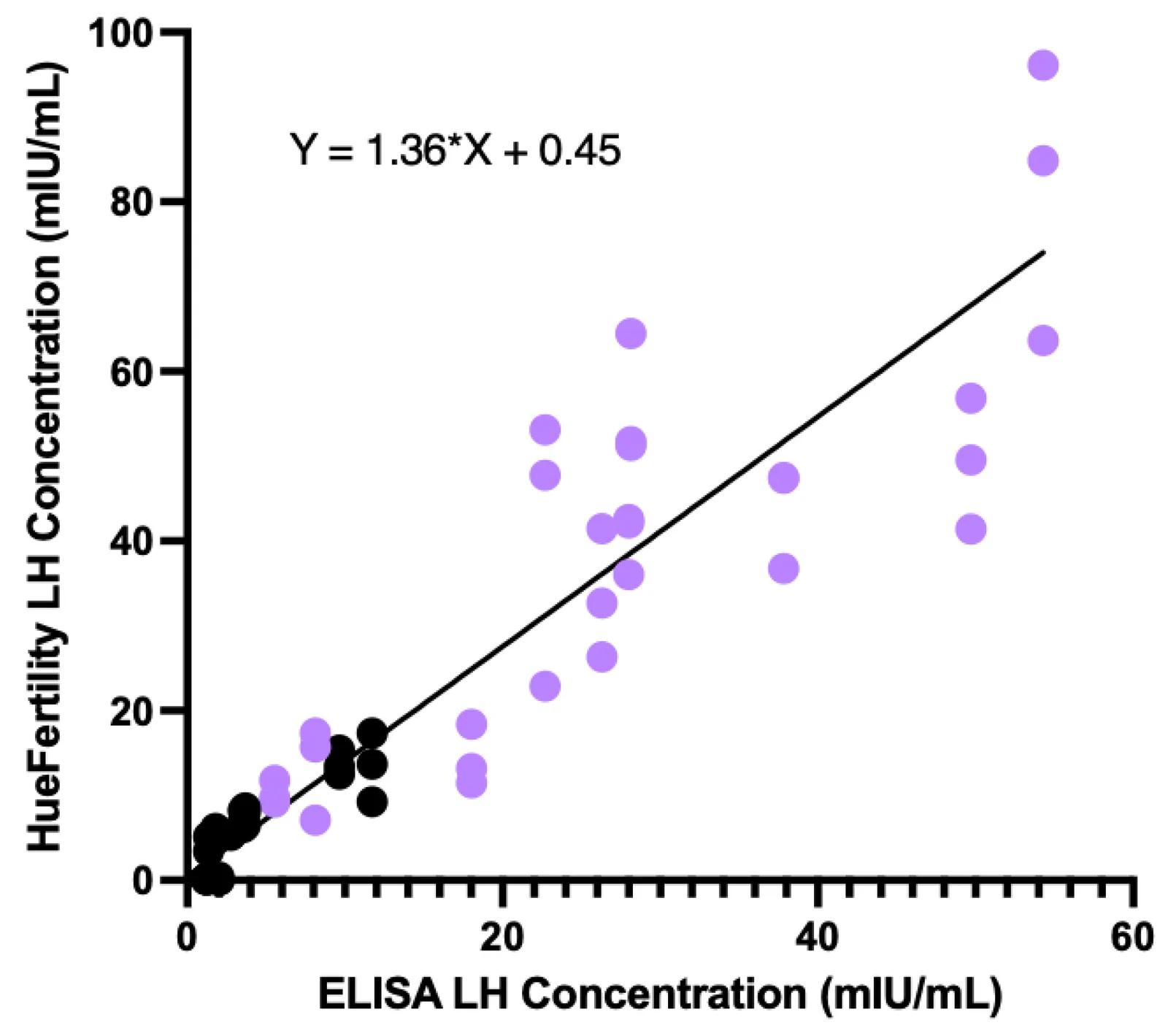
Native matrix validation. A Deming regression displaying slope and intercept. Black data points indicate native female urine samples and colored data points indicate LH-spiked female urine samples.

**Figure 8:**
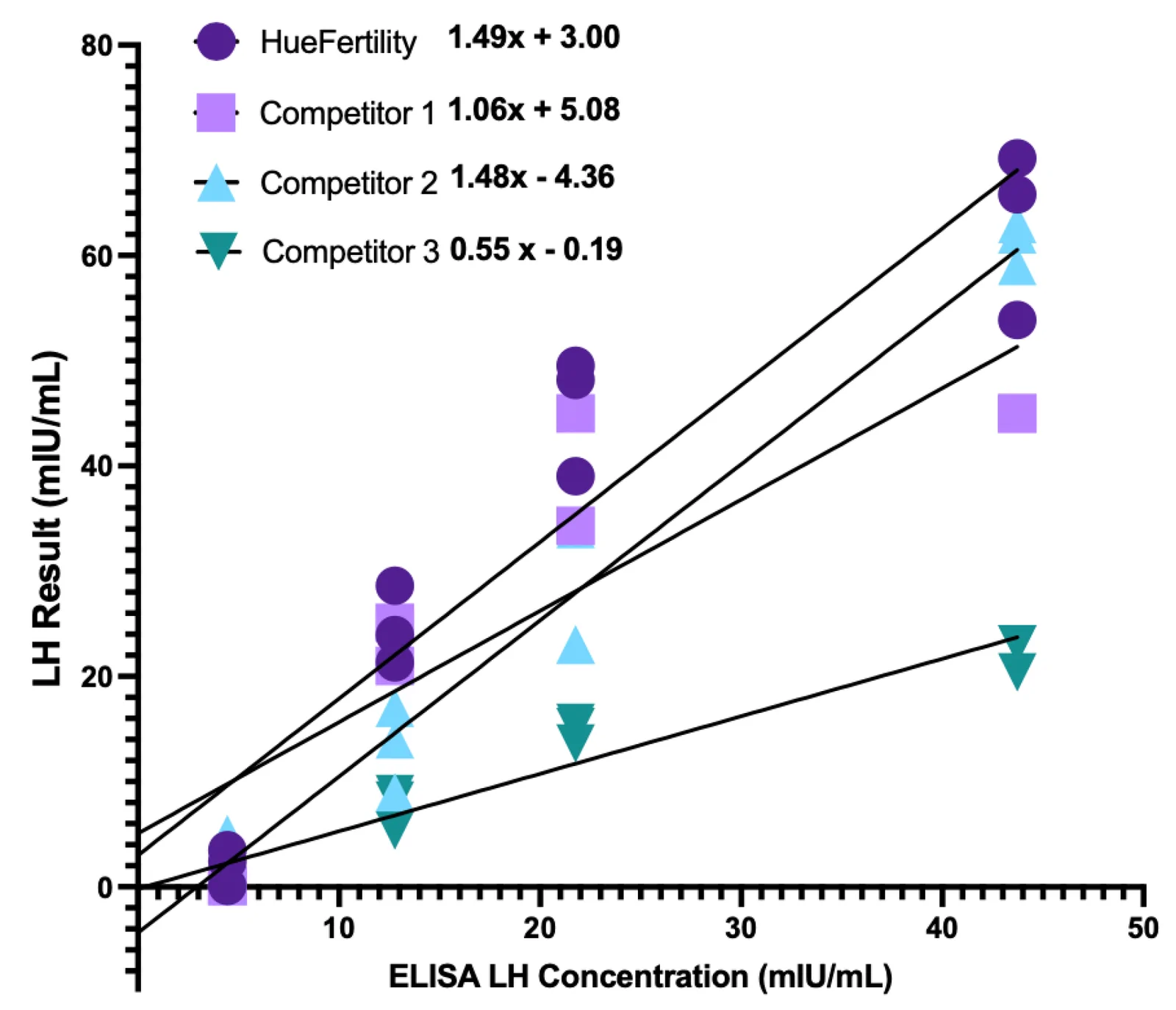
Competitor comparison study. Deming regression displaying slope and intercept across all devices.

**Table 1:** Distribution of physical strips and images across the training, model-selection validation, and independent evaluation sets by LH concentration. Each entry gives physical strips/images. All data were obtained using strips from a single reagent lot.

LH Concentration (mIU/mL)	Training	Validation	Independent Evaluation
0	9/31	3/11	9/9
5	9/27	3/9	3/6
10	9/27	3/9	6/9
15	9/31	3/14	3/6
20	9/31	3/11	9/9
25	9/27	3/9	6/9
40	9/31	3/11	3/6
50	9/32	3/10	8/9
60	9/31	3/11	3/6
70	9/27	3/9	9/9
80	9/27	3/9	3/6
90	9/27	3/9	3/6
100	9/27	3/9	6/9
Total	117/376	39/131	71/99

**Table 2:** Image distribution across phones and acquisition locations for each dataset. Phone and location columns are separate descriptions of the same images and are not additive. All data were obtained using strips from a single reagent lot.

Partition	Images	By iPhone Model	By Location
Training	376	11: 170; 13: 87; 16: 94; 17: 25	1: 142; 2: 117; 3: 117
Validation	131	11: 63; 13: 30; 16: 27; 17: 11	1: 51; 2: 40; 3: 40
Independent evaluation	99	11: 28; 13: 27; 16: 41; 17: 3	1: 49; 2: 34; 3: 16

**Table 3:** Mean difference between the control (no interferent) and spiked interferent samples (n = 10) with luteinizing hormone concentrations 0 mIU/mL (blank), 10 mIU/mL (low), and 50 mIU/mL (high).

Interferent	Mean Difference LH Blank	Mean Difference LH Low	Mean Difference LH High
FSH 50 mIU/mL	0.01	8.07	4.66
TSH 0.004 mIU/mL	−0.014	5.40	−0.044
β-hCG 25 mIU/mL	1.15	−7.33	0.50

**Table 4:** Analytical performance and value proposition of consumer urinary LH tests compared against reference ELISA.

	HueFertility	Competitor 1	Competitor 2	Competitor 3
Cost per test ($)	0.60	1.90	3.25	3.50
Reader cost ($)	0	0	170	100
Testing time (min)	8	10	16	10
Slope	1.49	1.06	1.48	0.55
95% CI (Slope)	1.04 to 1.94	0.41 to 1.71	1.37 to 1.60	0.43 to 0.66
Intercept	3.00	5.08	−4.36	−0.19
95% CI (Intercept)	−6.65 to 12.64	−11.55 to 21.70	−7.80 to −0.93	−2.93 to 2.55

## Data Availability

The raw data supporting the conclusions of this article will be made available by the authors on request.

## Supplementary Materials

The following supporting information can be downloaded at https://www.mdpi.com/article/10.3390/bios16100589/s1. Video S1: HueFertility Ovulation Workflow.
